# Preoperative identification of clinicopathological prognostic factors for relapse-free survival in clinical N1 non-small cell lung cancer: a retrospective single center-based study

**DOI:** 10.1186/s13019-020-01272-2

**Published:** 2020-08-28

**Authors:** Masato Aragaki, Tatsuya Kato, Aki Fujiwara-Kuroda, Yasuhiro Hida, Kichizo Kaga, Satoru Wakasa

**Affiliations:** Department of Cardiovascular and Thoracic Surgery, Hokkaido University Faculty of Medicine, Hokkaido University Graduate School of Medicine, West-7, North-15, Kita-ku, Sapporo, Hokkaido 060-8638 Japan

**Keywords:** Clinical N1, Maximum standardized uptake value, Multivariate analysis, Non-small cell lung cancer, Positron emission tomography

## Abstract

**Background:**

Given the difficulty in preoperatively diagnosing lymph node metastasis, patients with Stage I–III non-small cell lung cancer (NSCLC) are likely to be included in the clinical N1 (cN1) group. However, better treatment options might be selected through further stratification. This study aimed to identify preoperative clinicopathological prognostic and stratification factors for patients with cN1 NSCLC.

**Methods:**

This retrospective study evaluated 60 patients who were diagnosed with NSCLC during 2004–2014. Clinical nodal status had been evaluated using routine chest computed tomography (CT) and/or positron emission tomography (PET). To avoid biasing the fluorodeoxyglucose uptake values based on inter-institution or inter-model differences, we used only two PET systems (one PET system and one PET/CT system). Relapse-free survival (RFS) and overall survival (OS) were the primary study outcomes. The maximum standardized uptake value (SUVmax) was calculated for each tumor and categorized as low or high based on the median value. Patient sex, age, histology, tumor size, and tumor markers were also assessed.

**Results:**

Poor OS was associated with older age (*P* = 0.0159) and high SUVmax values (*P* = 0.0142). Poor RFS was associated with positive carcinoembryonic antigen (CEA) expression (*P* = 0.0035) and high SUVmax values (*P* = 0.015). Multivariate analyses confirmed that poor OS was independently predicted by older age (hazard ratio [HR] = 2.751, confidence interval [CI]: 1.300–5.822; *P* = 0.0081) and high SUVmax values (HR = 5.121, 95% CI: 1.759–14.910; *P* = 0.0027). Furthermore, poor RFS was independently predicted by positive CEA expression (HR = 2.376, 95% CI: 1.056–5.348; *P* = 0.0366) and high SUVmax values (HR = 2.789, 95% CI: 1.042–7.458; *P* = 0.0410). The primary tumor’s SUVmax value was also an independent prognostic factor for both OS and RFS.

**Conclusions:**

For patients with cN1 NSCLC, preoperative prognosis and stratification might be performed based on CEA expression, age, and the primary tumor’s SUVmax value. To enhance the prognostic value of the primary tumor’s SUVmax value, minimizing bias between facilities and models could lead to a more accurate prognostication.

## Background

Primary lung cancer is the leading cause of cancer-related deaths, with non-small cell lung cancer (NSCLC) accounting for approximately 80% of these cases [1]. Patients with lung cancer are often diagnosed at an advanced stage and experience fatal disease progression, despite improvements in surgical technique and chemoradiotherapy [1]. Thus, preoperative diagnosis and staging are important for lung cancer and greatly affect the selection of treatment strategies [2]. If surgical resection is possible, clinical Stage I NSCLC is typically treated with upfront surgery, while neo-adjuvant chemotherapy is recommended for clinical Stage III NSCLC [3–5]. Therefore, in resectable lung cancer cases, it is important to accurately diagnose the extent of lymph node metastasis, which is the major determinant of clinical staging, although this remains challenging [6–8].

The guidelines recommend primary surgical resection for clinical N1 (cN1) NSCLC, as well as adjuvant chemotherapy if nodal disease is confirmed postoperatively [9]. However, given the difficulty in preoperatively diagnosing lymph node metastasis, all patients with Stage I–III disease are likely to be provisionally included in the cN1 group. This can lead to suboptimal treatment based on a single treatment strategy in at least some of these cases. This approach may also explain the less-than-satisfactory results being reported during the treatment of cN1 cases [10]. The present study aimed to retrospectively evaluate cN1 NSCLC cases in an attempt to identify preoperative clinicopathological factors that might predict the prognosis and guide the selection of optimal treatment strategies.

## Patients and methods

### Patients

Between January 2000 and December 2014, 908 consecutive patients underwent surgery for NSCLC at our institution. Patients were clinically staged according to the seventh edition of the Union for International Cancer Control TNM classification [11]. Among these patients, we identified 78 patients (8.6%) with cN1 NSCLC based on preoperative computed tomography (CT) and/or positron emission tomography (PET), although 11 patients were excluded because they were diagnosed at other facilities. Among the 67 patients with cN1 NSCLC, we ultimately included 60 patients who had undergone complete anatomical resection of the involved segment, lobe, or lung, with mediastinal and hilar lymph node dissection, but without induction therapy. All patients provided informed consent for the procedure and provided written informed consent for institutional storage of their personal data in a scientific database. The medical ethics committee of the Hokkaido University School of Medicine approved the present study’s retrospective protocol (#019–0346), which was registered at Researchregistry.com (#5246).

### Diagnosis of cN1

Clinical nodal status was evaluated using routine chest CT and/or PET. Lymph nodes with a ≥ 10-mm short axis on chest CT or with asymmetric abnormal uptake on ^18^F-fluorodeoxyglucose (FDG) imaging were defined as being positive for lymph node metastasis. Between 2000 and 2008, we used an FDG-PET Siemens ECAT EXACT HR+ scanner (Siemens/CTI, Knoxville, TN, USA), while after 2009 we used an FDG PET/CT Biograph 64 TruePoint with TrueV PET/CT scanner (Siemens Japan, Tokyo).

### Prognostic factors

The relationships between preoperatively evaluable clinicopathological factors and the patients’ outcomes were examined. The relevant factors were considered sex (female vs. male), age (≥70 years vs. < 70 years), histology (adenocarcinoma vs. non-adenocarcinoma), tumor size (> 30 mm vs. ≤30 mm), and tumor marker levels with our institutional cut-off values (carcinoembryonic antigen [CEA, 5.0 ng/mL], squamous cell carcinoma antigen [SCC, 2.0 ng/mL], and CYFRA expression, 3.5 ng/mL). We also considered the prognostic value of the primary tumor’s maximum standardized uptake value (SUVmax).

### Statistical analyses

All statistical analyses were performed using JMP software (version 14.0; SAS Institute Inc., NC, USA). Inter-group comparisons were performed using the χ^2^ test or Fisher’s exact test, as appropriate. Survival outcomes were plotted using the Kaplan-Meier method and compared using the log-rank test. Overall survival was defined as the time from surgery until death, regardless of the cause of death. Relapse-free survival (RFS) was defined as the time from surgery until the first instance of recurrence or death. Univariate and multivariate Cox proportional hazards models were used to analyze the associations between clinicopathological factors and survival. Differences were considered statistically significant at two-sided *P* values of < 0.05.

## Results

### Patient characteristics

The patients’ characteristics and clinical findings are summarized in Table 1. The study included 46 men and 14 women with a median age of 70 years (range: 32–81). The tumors were classified according to the World Health Organization International Histological Classification of Tumors [12] as adenocarcinoma in 27 patients (45%), squamous cell carcinoma in 27 patients (45%), pleomorphic carcinoma in 3 patients (5%), adenosquamous carcinoma in 2 patients (3.3%), and large cell carcinoma in 1 patient (1.7%). The median tumor size was 35 mm (range: 13–88 mm), with clinical T classifications of T1 for 16 patients (26.7%), T2 for 29 patients (48.3%), T3 for 14 patients (23.3%), and T4 for 1 patient (1.7%). The preoperative clinical stages were stage IIA for 41 patients (68.3%), stage IIB for 4 patients (6.7%), and stage IIIA for 15 patients (25%). In terms of tumor markers, the median serum CEA value was 4.8 ng/mL (range: 1.5–152.2 ng/mL), the median serum SCC value was 0.8 ng/mL (range: 0.1–19.7 ng/mL), and the median serum CYFRA value was 2.48 ng/mL (range: 0.59–53.08 ng/mL). Given the difference between the FDG-PET instrument models, the original SUVmax values were analyzed without correction, which revealed median SUVmax values of 6 in the FDG-PET group (range: 0–22.78) and 11.45 in the FDG-PET/CT group (range: 1.90–52.60). The surgical approaches were video-assisted thoracic surgery (VATS) for 23 patients (38.3%), standard open thoracic surgery for 20 patients (33.3%), and conversion from VATS to open surgery for 17 patients (28.3%). The resections involved lobectomy for 51 patients (85%), pneumonectomy for 7 patients (11.7%), and segmentectomy for 2 patients. Reconstruction of the pulmonary artery or bronchus was added for 6 of the 51 patients who had undergone lobectomy.

**Table 1:** Characteristics of the patients with clinical N1 non-small cell lung cancer

	Number
Sex	
Male	46
Female	14
Age (years)	
Median	70
Range	32–81
Histological type	
Adenocarcinoma	27
Squamous cell carcinoma	27
Pleomorphic carcinoma	3
Adenosquamous cell carcinoma	2
Large cell carcinoma	1
Size of primary tumor (mm)	
Median	35
Range	13–88
Clinical T status	
T1	16
T2	29
T3	14
T4	1
CEA (ng/mL)	
Median	4.8
Range	1.5–152.2
SCC (ng/mL)	
Median	0.8
Range	0.0–19.7
CYFRA (ng/mL)	
Median	2.48
Range	0.59–53.08
FDG-PET	
Number of patients	35
Median of SUVmax	6
Range of SUVmax	0–22.78
FDG-PET/CT	
Number of patients	25
Median of SUVmax	11.45
Range of SUVmax	1.90–52.60
Surgical approach	
VATS	23
Thoracotomy	20
Conversion (VATS to thoracotomy)	17
Operative procedure	
Pneumonectomy	7
Lobectomy	51
+ bronchoplasty	3
+ angioplasty	1
+ bronchoplasty & angioplasty	2
Segmentectomy	2
Pathological T status	
pT1	15
pT2	29
pT3	14
pT4	2
Pathological N status	
pN0	23
pN1	22
pN2	15
Pathological stage	
IA	4
IB	12
IIA	14
IIB	6
IIIA	23
IIIB	1

### Pathological diagnoses

The postoperative pathological tumor diagnoses were pT1 for 15 patients (25%), pT2 for 29 patients (48.3%), pT3 for 14 patients (23.3%), and pT4 for 2 patients (3.3%). Similar to the clinical diagnoses, the most common pathological diagnosis was pT2. The pathological nodal diagnoses revealed correct preoperative diagnoses of N1 for 22 patients (36.7%), N0 for 23 patients (38.3%), and N2 for 15 patients (25%). Twenty-four patients (40%) were postoperatively diagnosed with stage III disease (Table 1).

### Survival rate

**Fig. 1:**
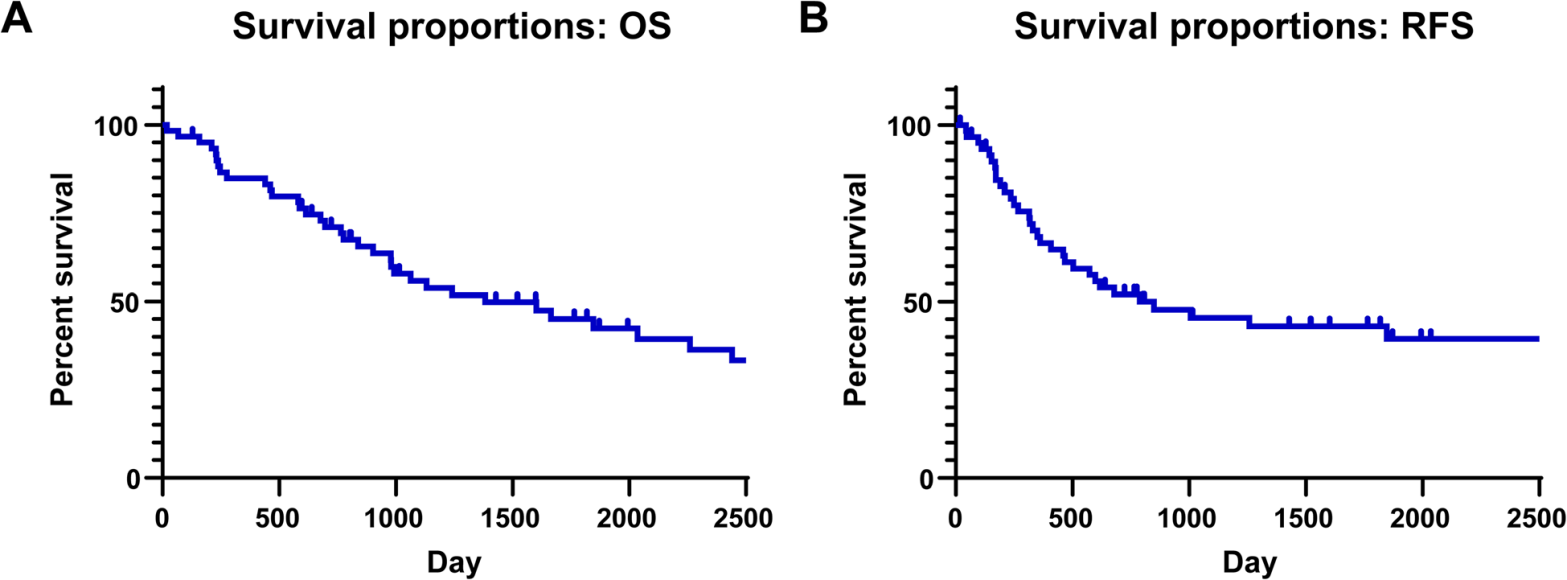
Overall survival and relapse-free survival curves after complete resection of cN1 non-small cell lung cancer. **a** The overall survival (OS) curve and 5-year survival rate among all patients (5-year OS rate: 45.1%), **b** The relapse-free survival (RFS) curve and 5-year survival rate among all patients (5-year RFS rate: 43.0%). *P < 0.05

**Fig. 2:**
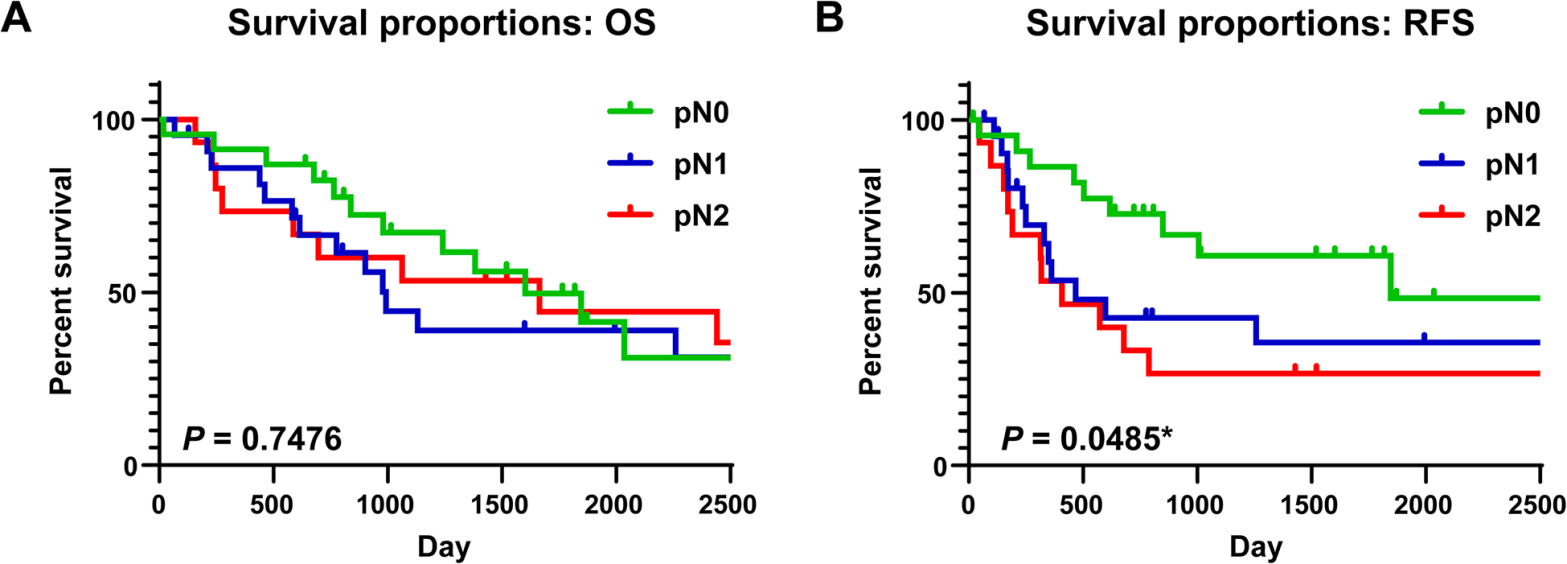
Overall survival and relapse-free survival curves according to pathological N status. **a** The overall survival (OS) curves and 5-year survival rates according to pathological N status (5-year OS rate: pN0 = 49.8%, pN1 = 39.1%, pN2 = 44.4%), **b** The relapse-free survival **(**RFS) curves and 5-year survival rates according to pathological N status (5-year RFS rate: pN0 = 60.1%, pN1 = 35.6%, pN2 = 26.7%). *P < 0.05

Among the included cN1 cases, the 5-year OS rate was 45.1% and the 5-year RFS rate was 43.0% (Fig. 1). When we considered the pathological diagnoses, no significant difference was observed when we compared the 5-year OS rates between the pN0 (49.8%), pN1 (44.4%), and pN2 groups (44.4%, *P* = 0.7476). However, we detected a significant difference in the 5-year RFS rates according to the pathological nodal classification (pN0: 60.1%, pN1: 35.6%, pN2: 26.7%, *P* = 0.0485) (Fig. 2).

### Clinicopathological factors for predicting OS and RFS

The prognostic values of the clinicopathological factors are shown in Table 2. We found that there was a large difference in the median SUVmax values that were determined using PET and PET/CT (6.0 vs. 11.45). Thus, we chose to use the raw data without correction because the SUVmax value is influenced by various parameters. Patients with values above the median SUVmax values for PET or PET/CT were assigned to the high-value group, and patients with lower values were assigned to the low-value group.

**Table 2:** Clinicopathological factors for predicting disease-free survival

Variable	Unfavorable/favorable	Number
Sex	Female vs. male	14 / 46
Age	≥70 vs. < 70 years	32 / 28
Histology	Adenocarcinoma vs. others	27 / 33
Tumor size	> 30 vs. ≤30 mm	41 / 19
CEA expression	≥5.0 vs. < 5.0 ng/mL	30 / 30
Primary tumor SUVmax	High vs. low	29 / 31

**Fig. 3:**
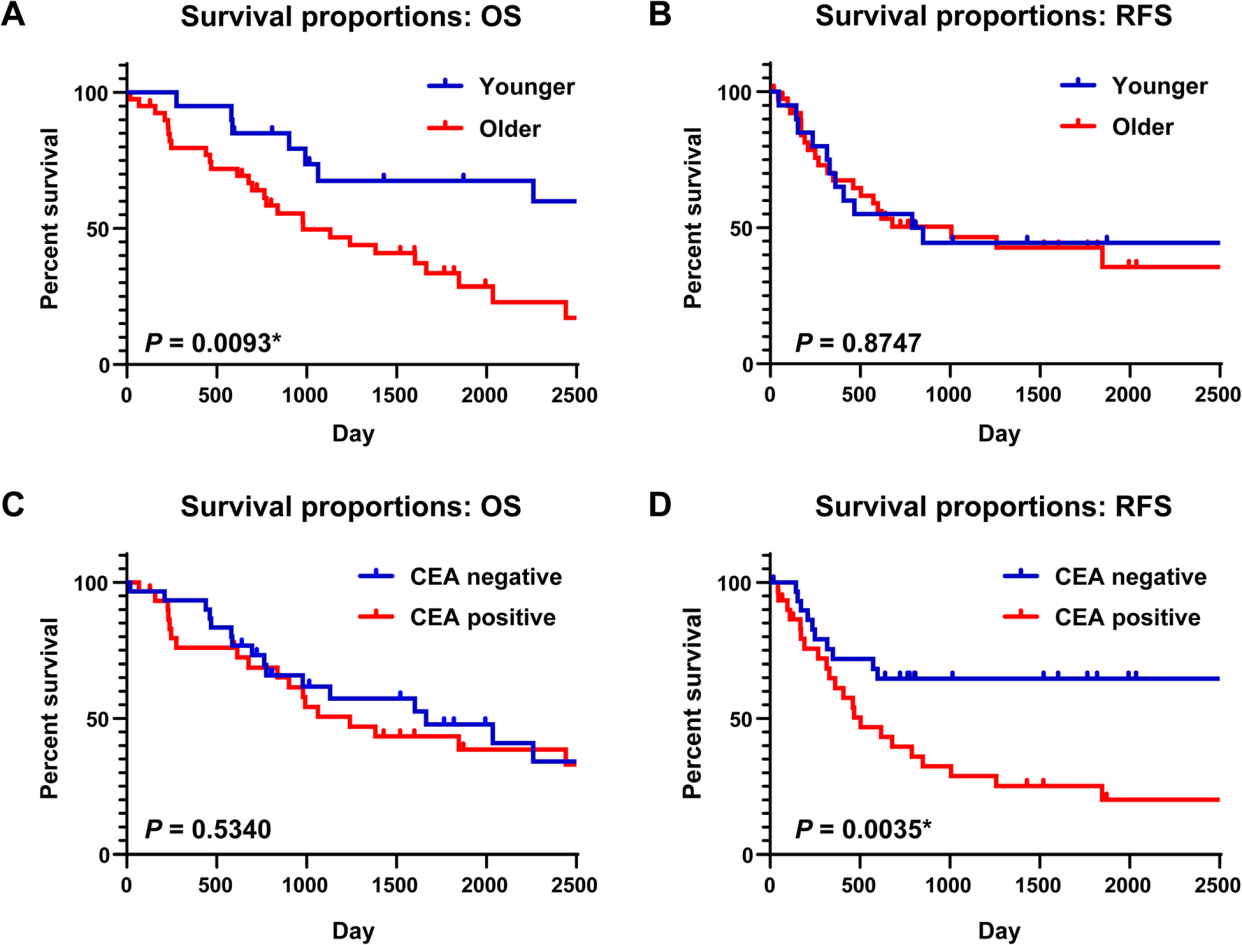
Survival curves stratified according to age and carcinoembryonic antigen expression. **a**, **b** The overall survival (OS) and relapse-free survival (RFS) curves with 5-year survival rates are shown according to ages of ≥70 years and < 70 years (5-year OS rate: 30.9% vs. 61.5%, log-rank *P* = 0.0159; 5-year RFS rate: 29.8% vs. 41.7%, log-rank *P* = 0.9543). **c**, **d** The OS and RFS curves with 5-year survival rates according to normal and abnormal expression of carcinoembryonic antigen (CEA; 5-year OS rate: 43.4% vs. 47.8%, log-rank *P* = 0.534; 5-year RFS rate: 25.2% vs. 64.7%, log-rank *P* = 0.0035). *P < 0.05

**Fig. 4:**
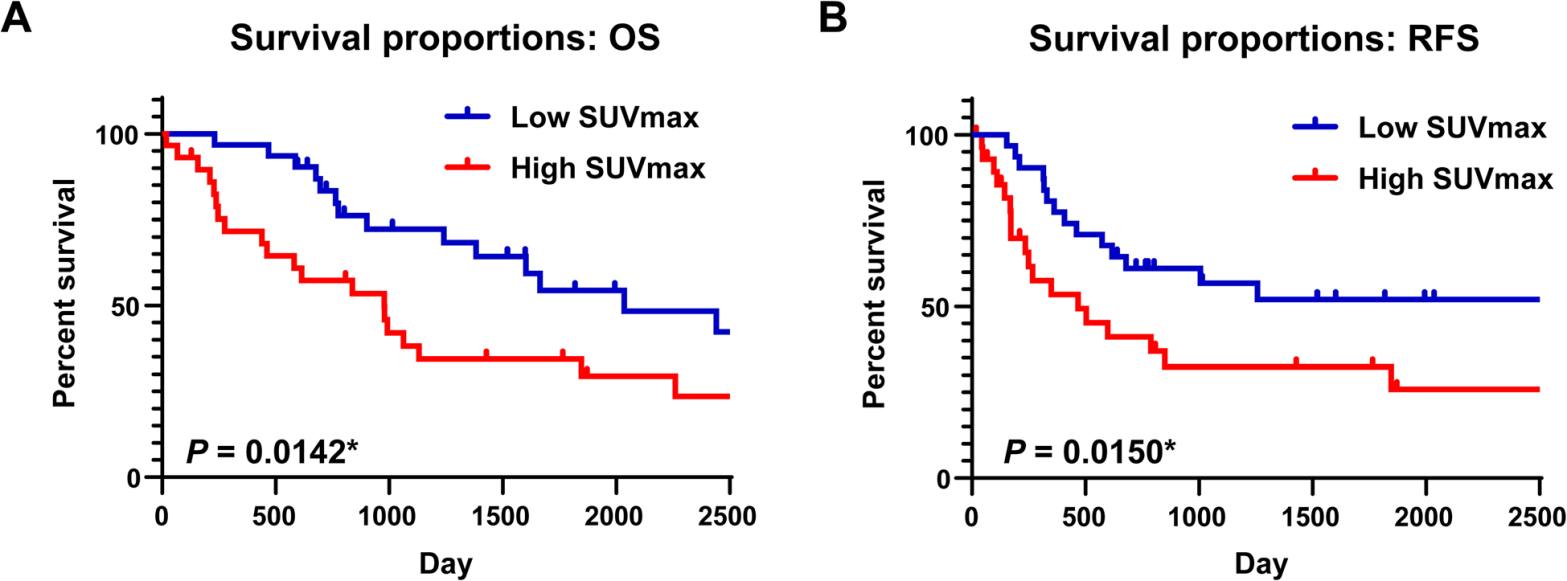
Survival curves stratified according to SUVmax values. Kaplan-Meier analysis of survival among cN1 NSCLC patients. **a**, **b** The overall survival (OS) and relapse-free survival (RFS) according to high and low SUVmax values for the primary tumor (5-year OS rate: 34.4% vs. 54.4%, log-rank *P* = 0.0142; 5-year RFS rate: 32.4% vs. 52.0%, log-rank *P* = 0.015). *P < 0.05

The OS and RFS outcomes were analyzed according to the clinicopathological factors. Based on the log-rank test, age was significantly associated with poor OS (*P* = 0.0159) but not with poor RFS (*P* = 0.9543) (Fig. 3a & b). The CEA value was not significantly associated with poor OS (*P* = 0.534), but was significantly associated with poor RFS (*P* = 0.0035) (Fig. 3c & d). However, the SCC and CYRA values were not significantly associated with OS or RFS (SCC, OS: *P* = 0.534, RFS: *P* = 0.0035; CYFRA, OS: *P* = 0.534, RFS: *P* = 0.0035) (Supplementary Fig. 1). The primary tumor’s SUVmax value was significantly associated with both poor OS (*P* = 0.0142) and poor RFS (*P* = 0.015) (Fig. 4). No significant differences in OS or RFS were observed when we compared sex (OS: *P* = 0.8516, RFS: *P* = 0.5446), histology (OS: *P* = 0.6415, RFS: *P* = 0.5027), and tumor size (OS: *P* = 0.7621, RFS: *P* = 0.871) (Supplementary Fig. 1).

The univariate Cox proportional hazards model revealed similar results to those obtained with the Kaplan-Meier analyses. The expression of CEA was used as a representative tumor marker, because only CEA was significantly associated with prognosis based on the Kaplan-Meier analysis. Poor OS was significantly associated with older age (hazard ratio [HR]: 2.369, 95% confidence interval [CI]: 1.152–4.870; *P* = 0.019) and high SUVmax values for the primary tumor (HR: 2.284, 95% CI: 1.159–4.502; *P* = 0.017). Poor RFS was associated with abnormal preoperative serum CEA levels (HR: 2.901, 95% CI: 1.374–6.127; *P* = 0.005) and high SUVmax values for the primary tumor (HR: 2.312, 95% CI: 1.155–4.630; *P* = 0.018). The multivariate Cox proportional hazard model also confirmed that a high SUVmax value for the primary tumor independently predicted poor OS (HR: 5.121, 95% CI: 1.759–14.910; *P* = 0.003) and poor RFS (HR: 2.789, 95% CI: 1.042–7.458; *P* = 0.041) in patients with cN1 NSCLC. In addition, age independently predicted poor OS (HR: 2.751, 95% CI: 1.300–5.822; *P* = 0.008) and CEA expression independently predicted poor RFS (HR: 2.376, 95% CI: 1.056–5.348; *P* = 0.037) (Table 3). We also evaluated whether the SUVmax value was associated with the various clinicopathological factors, including pN1 status. The results revealed that a high SUVmax was significantly associated with non-adenocarcinoma type (*P* = 0.011) and tumor size of ≥30 mm (*P* = 0.001), but not pN status (*P* = 0.211) (Table 4).

**Table 3:** Cox proportional hazards analysis of prognostic factors in patients with cN1 disease

	Unfavorable vs. favorable	OS			RFS		
		HR	95% CI	*P*-value	HR	95% CI	*P*-value
Univariate analysis							
Sex	Female vs. male	1.075	0.502–2.305	0.8517	1.269	0.586–2.750	0.5455
Age	≥70 vs. < 70 years	2.369	1.152–4.870	0.0190*	1.020	0.510–1.960	0.9543
Histology	Adenocarcinoma vs. others	1.171	0.602–2.277	0.6419	1.265	0.635–2.520	0.5037
Tumor size	> 30 vs. ≤30 mm	0.892	0.427–1.865	0.7622	1.066	0.495–2.296	0.8710
CEA	≥5.0 vs. < 5.0 ng/mL	1.235	0.634–2.405	0.5348	2.901	1.374–6.127	0.0052*
Primary tumor SUVmax	High vs. low	2.284	1.159–4.502	0.0171*	2.312	1.155–4.630	0.0180*
Multivariate analysis							
Sex	Female vs. male	1.315	0.556–3.276	0.5564	1.018	0.430–2.413	0.9669
Age	≥70 vs. < 70 years	2.751	1.300–5.822	0.0081*	1.130	0.545–2.343	0.7419
Histology	Adenocarcinoma vs. others	1.970	0.754–5.149	0.1668	1.353	0.575–3.181	0.4884
Tumor size	> 30 vs. ≤30 mm	1.989	0.603–6.565	0.2589	0.523	0.186–1.475	0.2205
CEA	≥5.0 vs. < 5.0 ng/mL	1.039	0.504–2.143	0.9170	2.376	1.056–5.348	0.0366*
Primary tumor SUVmax	High vs. low	5.121	1.759–14.910	0.0027*	2.789	1.042–7.458	0.0410*

*HR* Hazard ratio, *CI* Confidence interval. *P < 0.05

**Table 4:** Differences in clinicopathological factors according to the primary tumor’s SUVmax

	Total (*n* = 60)	Primary tumor’s SUVmax		*P* value
		High	Low	
Sex				
Male	46	23	23	1.000
Female	14	6	8	
Age				
< 70 years	28	14	14	0.7961
≥ 70 years	32	15	17	
Histological type				
Adenocarcinoma	27	8	19	0.0108*
Non-adenocarcinoma	33	21	12	
Tumor size				
≤ 30 mm	19	3	16	< 0.0001*
> 30 mm	41	26	15	
CEA				
< 5.0 ng/mL	30	11	19	0.1205
≥ 5.0 ng/mL	30	18	12	
Pathological N status ng/mL				
pN0	23	9	14	0.2106
pN1	22	14	8	
pN2	15	6	9	

*P < 0.05

## Discussion

The present study demonstrated that the prognoses of patients with cN1 NSCLC were associated with age, the CEA value, and the primary tumor’s SUVmax value. The primary tumor’s SUVmax value was also an independent prognostic factor for both OS and RFS, which is consistent with previous reports regarding the associations between FDG uptake and tumor malignancy [13–16]. Clinical and experimental studies have indicated that FDG accumulation during PET examination was correlated with tumor growth rate, cell density, and cell differentiation [17–19]. These results support our finding that a high value for the primary tumor’s SUVmax may predict high-grade disease and a poor prognosis. Furthermore, the associations of SUVmax with histological type and tumor size may reflect the malignant potential of the primary tumor. Nevertheless, it appears that the SUVmax value’s relationship with prognosis is not solely explained by a correlation with the pN status.

There can be some variations in the FDG uptake values based on inter-institution or inter-model differences between the PET instruments, and it is possible that its true effectiveness as a prognostic marker cannot be accurately assessed without considering these differences. For example, it would be appropriate to consider the SUVmax value for a separate group when a phantom is used, and to also consider the PET model or facility [20–23]. Furthermore, SUVmax values can vary according to the instrument model, size of the patient’s body, and the presence or absence of diabetic complications [24–26]. Moreover, different facilities use different PET protocols (e.g., image acquisition timing and the use of one or two scans), which also complicates the analysis of data from multiple facilities. Therefore, we examined the original SUVmax values without correction for each instrument model. It would be ideal to conduct a study using the same PET instruments, although it would be difficult to accumulate an appropriate number of cN1 cases. Thus, we believe that a single-center analysis using only two PET instrument models may be a useful starting point. Finally, because FDG integration was significantly different between the instrument models, we categorized the SUVmax values as high or low using the median values from the time periods when each instrument was used (2000–2008 and 2009–present).

While analyzing the cN1 NSCLC cases, we encountered two problems that should be considered. The first problem was the accuracy of the preoperative diagnosis, and the second problem was the recommended treatment strategy. An accurate preoperative assessment is critical for selecting the most suitable treatment for NSCLC patients [27]. However, despite advances in diagnostic CT and FDG/PET-CT, over-diagnosis and under-diagnosis of nodal metastasis can occur easily because cN1 disease is a marginal stage for surgery. It has been reported that 19–30% of patients with a preoperative diagnosis of cN1 were diagnosed with pN2 disease after the surgery [6, 10, 11, 28], and our findings revealed a similar result (15 patients [25%] with preoperative cN1 disease were found to have pN2 disease). A meta-analysis has indicated that endobronchial ultrasound-guided transbronchial needle aspiration (EBUS-TBNA) is a potentially useful technique that can provide a sensitivity of 88–94% for mediastinal staging of lung cancer [29]. However, the use of EBUS-TBNA to diagnose lymph node metastasis is relatively new, and would likely be used in only a small proportion of cases, so this factor was not included in the present study.

Several randomized trials and meta-analyses have shown survival benefits for adjuvant chemotherapy after surgery in stage II–III NSCLC patients [30–32]. However, some reports have indicated that induction chemotherapy was not associated with improved survival in cN1 NSCLC patients [33, 34]. Therefore, the current guidelines for patients with cN1 NSCLC recommend a surgery-first strategy, followed by adjuvant chemotherapy for patients who have pathologically confirmed nodal metastasis [35]. Furthermore, the clinical stage is determined based on the tumor size, the lymph node metastasis, and the distant metastasis diagnosis at the time of each examination. However, cN1 NSCLC patients are a heterogeneous population and we believe that a more personalized treatment strategy might be useful in this population. Thus, we hope to guide treatment selection based on preoperatively evaluable parameters, as we believe that malignant potential (e.g., measured based on tumor growth rate) is not reflected in the current clinical staging of NSCLC. We hypothesize that cN1 NSCLC patients might be stratified based on preoperative factors that reflect the tumor’s malignant potential, in additional to other standard clinical staging factors, which might guide treatment selection before the patient undergoes surgery.

The present study has several limitations that should be considered. First, the small sample size and retrospective nature of the study are prone to bias. However, we hope to collect additional cases and potentially incorporate more accurate preoperative diagnoses using the EBUS-TBNA technique. Second, the cut-off value for SUVmax varied according to the PET instrument model, although previous studies have also used values from multiple facilities/models, which might have obscured the association between SUVmax and prognosis.

## Conclusion

We retrospectively reviewed the clinicopathological characteristics of patients with cN1 NSCLC who underwent surgical resection. Although it is difficult to accurately and preoperatively diagnose nodal metastasis using CT and/or PET, our results suggest that the primary tumor’s SUVmax value may be preoperatively used for prognostication in cN1 NSCLC cases. Thus, the SUVmax value might be useful for patient stratification and treatment selection. However, further studies are needed to clarify the differences in FDG accumulation between facilities and/or instrument models, in order to confirm whether this marker is reliable in this setting. Nevertheless, if FDG accumulation is found to be a useful prognostic factor, it might help preoperatively guide the selection of newer therapeutic strategies, including adjuvant chemotherapy, for patients with cN1 NSCLC.

## Supplementary information

**Additional file 1: Figure S1.** Survival curves stratified according to various clinicopathological factors. **a, b** The overall survival (OS) and relapse-free survival (RFS) curves with 5-year survival rates according to female and male sex (5-year OS rate: 42.9% vs. 45.5%, log-rank *P* = 0.8516; 5-year RFS rate: 32.1% vs. 46.4%, log rank *P* = 0.5446). **c, d** The OS and RFS curves with 5-year survival rates according to adenocarcinoma and non-adenocarcinoma classification (5-year OS rate: 43.4% vs. 46.4%, log-rank *P* = 0.6415; 5-year RFS rate: 37.1% vs. 48.7%, log-rank *P* = 0.5027). **e, f** The OS and RFS curves with 5-year survival rates according to tumor diameters of ≥30 mm and < 30 mm (5-year OS rate: 47.3% vs. 38.9%, log-rank *P* = 0.7621; 5-year RFS rate: 44.6% vs. 37.5%, log-rank *P* = 0.871). **g, h** The OS and RFS curves with 5-year survival rates according to positive and negative expression of squamous cell carcinoma antigen (SCC; 5-year OS rate: 28.1% vs. 48.0%, log-rank *P* = 0.1254; 5-year RFS rate: 34.2% vs. 35.8%, log-rank *P* = 0.2338). **i, j** The OS and RFS curves and 5-year survival rates according to positive and negative expression of CYFRA (5-year OS rate: 49.2% vs. 43.0%, log-rank *P* = 0.7879; 5-year RFS rate: 50.4% vs. 28.7%, log-rank *P* = 0.5767). *: *P* < 0.05.

## Data Availability

The study protocol was registered at Researchregistry.com (#5246). The datasets used and/or analyzed during the current study are available from the corresponding author upon reasonable request.

## Abbreviations

NSCLC: Non-small cell lung cancer
cN1: Clinical N1
CT: Computed tomography
PET: Positron emission tomography
RFS: Relapse-free survival
OS: Overall survival
SUVmax: Maximum standardized uptake value
CEA: Carcinoembryonic antigen
HR: Hazard ratio
FDG: ^18^F-fluorodeoxyglucose
VATS: Video-assisted thoracic surgery
EBUS-TBNA: Endobronchial ultrasound-guided transbronchial needle aspiration
