# Supplementary figures and images for: Preoperative identification of clinicopathological prognostic factors for relapse-free survival in clinical N1 non-small cell lung cancer: a retrospective single center-based study
Source: J Cardiothorac Surg. 2020 Aug 28;15:229. doi: 10.1186/s13019-020-01272-2 (PMC7456382; doi:10.1186/s13019-020-01272-2)

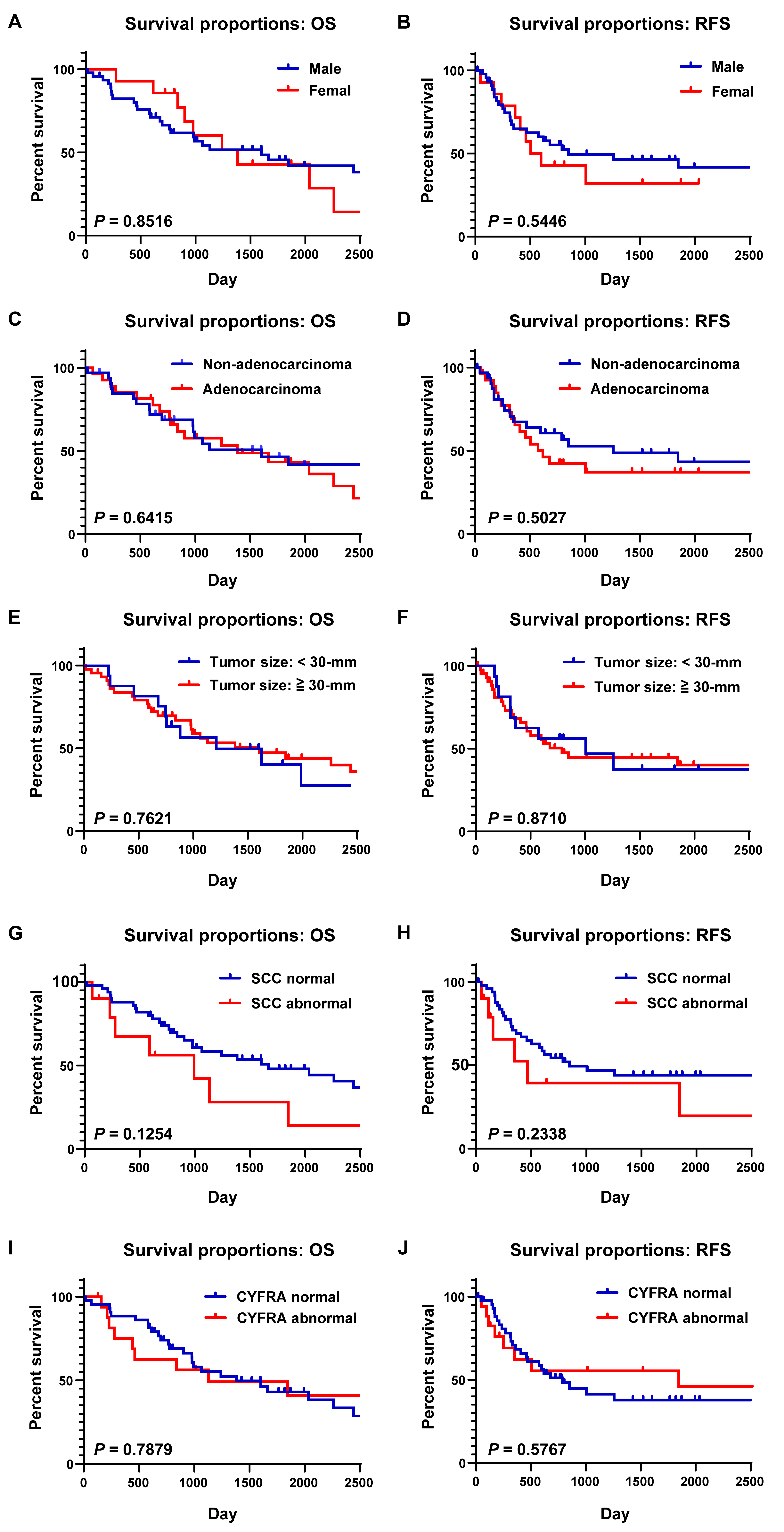

Supplement: Supplementary file 1 — Additional file 1: Figure S1. Survival curves stratified according to various clinicopathological factors. a, b The overall survival (OS) and relapse-free survival (RFS) curves with 5-year survival rates according to female and male sex (5-year OS rate: 42.9% vs. 45.5%, log-rank P = 0.8516; 5-year RFS rate: 32.1% vs. 46.4%, log rank P = 0.5446). c, d The OS and RFS curves with 5-year survival rates according to adenocarcinoma and non-adenocarcinoma classification (5-year OS rate: 43.4% vs. 46.4%, log-rank P = 0.6415; 5-year RFS rate: 37.1% vs. 48.7%, log-rank P = 0.5027). e, f The OS and RFS curves with 5-year survival rates according to tumor diameters of ≥30 mm and < 30 mm (5-year OS rate: 47.3% vs. 38.9%, log-rank P = 0.7621; 5-year RFS rate: 44.6% vs. 37.5%, log-rank P = 0.871). g, h The OS and RFS curves with 5-year survival rates according to positive and negative expression of squamous cell carcinoma antigen (SCC; 5-year OS rate: 28.1% vs. 48.0%, log-rank P = 0.1254; 5-year RFS rate: 34.2% vs. 35.8%, log-rank P = 0.2338). i, j The OS and RFS curves and 5-year survival rates according to positive and negative expression of CYFRA (5-year OS rate: 49.2% vs. 43.0%, log-rank P = 0.7879; 5-year RFS rate: 50.4% vs. 28.7%, log-rank P = 0.5767). *: P < 0.05. [file 13019_2020_1272_MOESM1_ESM.tif]
